# Nitrogen addition has negative effects on bacterial diversity and stability, but simulated grazing mitigates these effects

**DOI:** 10.3389/fmicb.2026.1747173

**Published:** 2026-02-17

**Authors:** Zeyu Liu, Yiqiang Dong, Anjing Jiang, Zongjiu Sun, Yue Wu, Yaxin Lei, Xingyun Shan, Kai Wu

**Affiliations:** 1 College of Grassland Science, Xinjiang Agricultural University, Ürümqi, China; 2 Key Laboratory of Grassland Resources and Ecology, Ürümqi, Xinjiang, China; 3 Key Laboratory of Grassland Resources and Ecology of Western Arid Region, Ministry of Education, Ürümqi, China

**Keywords:** grassland, microbial co-occurrence network, microbial diversity, nitrogen addition, simulated grazing

## Abstract

**Introduction:**

Nitrogen addition and grazing, as common management tools in grasslands, alter the structure and function of soil microbial communities and have far-reaching effects on grassland ecosystems. However, the mechanisms by which nitrogen addition and grazing regulate the diversity and stability of soil microbial communities remain insufficiently understood.

**Methods:**

In this study, a field experiment was conducted in the temperate desert grassland of Xinjiang, combining nitrogen addition treatments with simulated grazing to investigate the response mechanisms of soil microbial communities to nitrogen addition and simulated grazing. The regulation of soil microbial community diversity and stability under the combined effects of nitrogen addition and grazing was examined by using mowing to simulate aboveground vegetation disturbance.

**Results:**

The results showed that inorganic nitrogen (nitrate and ammonium nitrogen) was a key factor driving nitrogen-induced changes in microbial community structure, increasing the availability of soil nitrogen. Moderate nitrogen addition promoted bacterial community diversity, whereas excessive nitrogen input weakened this effect and reduced bacterial community complexity and co-occurrence network stability. Simulated grazing enhanced organic nitrogen catabolism through increased leucine aminopeptidase activity, thereby stabilizing bacterial community interactions under nitrogen-enriched conditions and alleviating the negative effects of nitrogen addition.

**Discussion:**

These results indicate that grazing can buffer nitrogen-induced destabilization of soil microbial communities and highlight its role in maintaining microbial functional stability in grassland ecosystems under increasing nitrogen deposition.

## 1 Introduction

Soil microorganisms play a central role in numerous processes within ecosystems. They serve not only as vital biological regulators of plant-available nutrients in ecosystems (Wang et al., 2021; Chen et al., 2024; Fang et al., 2025; Islam et al., 2020), but also participate in regulating key factors that maintain ecosystem stability (Li et al., 2025). Among these, bacteria and fungi, as key components of soil microorganisms, play vital roles in the cycling of soil organic matter and accelerating nutrient mineralization (Sokol et al., 2022). Nitrogen nutrients are typically limited in terrestrial ecosystems (Elser et al., 2007). Research indicates that nitrogen limitation adversely affects soil physicochemical properties (Fu et al., 2019), while nitrogen addition remains the most effective method for enhancing soil fertility. Research by Yue et al. (2017) indicates that nitrogen addition accelerates organic matter decomposition, leading to increased levels of available nitrogen and dissolved organic carbon in soil. The rise in available nitrogen content may alter the structure and function of soil microbial communities, thereby influencing biogeochemical cycles in terrestrial ecosystems (Chen et al., 2017).

Grassland ecosystems are among China’s largest terrestrial ecosystems, playing a vital role in soil and water conservation, windbreak and sand fixation, and biodiversity protection (Edwards et al., 2025). Nitrogen enrichment events influence grassland ecosystems by altering the nitrogen cycle, thereby exerting effects on vegetation, soil properties, and microbial communities (Zhang et al., 2022). Research indicates that excessive nitrogen inputs not only reduce soil microbial biomass in grasslands, leading to alterations in soil microbial community composition and enzyme activity (Ramirez et al., 2010), but also modify soil microbial diversity and richness (Ying et al., 2017). Both Wang et al. (2018) and Yang et al. (2020) showed that N addition significantly reduced soil microbial diversity in grassland ecosystems, which was associated with N-addition-induced soil acidification and reduction in soil microbial biomass, and that soil fungal diversity was more sensitive to N addition compared to soil bacterial diversity. However, not all of the negative impacts of nitrogen addition on soil ecosystems are negative. Yang et al. (2022) showed that nitrogen addition reduced bacterial and fungal diversity in the soil but increased soil organic carbon and microbial biomass carbon, and that this carbon accumulation came at the cost of weakening the link between it and microbial diversity. These findings suggest that nitrogen-induced changes in soil microbial communities affect nutrient cycling and ecosystem stability in grassland ecosystems, and provide theoretical references for further exploration of the negative effects of nitrogen enrichment on microbial communities.

With the exception of nitrogen addition, grazing is the most cost-effective management tool in grassland ecosystems (Julihaiti et al., 2025). Grazing determines the structure and function of grassland ecosystems by regulating the interrelationships between plants, soils and microorganisms (Ma et al., 2024; Sun et al., 2021). Studies have shown that moderate grazing helps increase soil organic matter, total nitrogen, and total phosphorus content, whereas overgrazing inhibits the accumulation of these nutrients, further altering the structure of soil microbial communities (Zhan et al., 2020). However, reports on the effects of grazing on soil microorganisms varied across studies, depending on the type of grassland, the intensity of grazing, and the different microbial community. For example, Wang et al. (2024b) found that grazing significantly reduced the bacterial diversity of desert steppe, but had no significant effect on the fungal community, while Jing et al. (2023) found that high grazing intensity significantly altered the network complexity of soil microbial communities in alpine meadows, and that the complexity of the fungal network gradually decreased with the increase of grazing intensity. Currently, 90% of grasslands in China are experiencing varying degrees of degradation, with long-term overgrazing disrupting the nutrient cycling processes of grassland ecosystems by altering soil microbial community structure (e.g., reducing fungal abundance and disturbing bacterial diversity) (Xun et al., 2018; Jing et al., 2023). Therefore, appropriate grazing management is essential for ensuring the sustainability, restoration, and conservation of its ecosystem.

As an important component of Xinjiang’s grasslands, temperate desert steppe ecosystems are fragile and highly susceptible to disturbances from human activities, particularly nitrogen deposition and grazing (Han et al., 2025). Previous studies have extensively investigated the effects of nitrogen application alone or grazing on soil microbial communities, confirming that both factors can alter microbial diversity, community composition and nutrient-related processes. However, most studies have focused on the effects of a single factor, and the interactive mechanisms by which nitrogen addition and grazing co-regulate microbial community diversity and stability remain unclear. To disentangle the effects of aboveground biomass removal from other grazing-associated disturbances, mowing was applied in this study as a simulated grazing treatment to mimic grazing-induced changes in vegetation structure. Accordingly, a field experiment with different nitrogen addition levels and simulated grazing treatments was conducted in a temperate desert steppe to investigate their individual and interactive effects on soil microbial communities. Specifically, this study aims to address whether and how grazing mitigates nitrogen-induced changes in soil microbial diversity and stability within temperate desert steppes. Based on this overarching question, we propose the following related hypotheses: (1) microbial communities exhibit a nonlinear response to increasing nitrogen addition; (2) inorganic nitrogen is the primary driver of microbial community diversity changes under nitrogen addition; and (3) grazing mitigates the negative effects of excessive nitrogen addition on microbial communities.

## 2 Materials and methods

### 2.1 Location of study area

**Figure 1:**
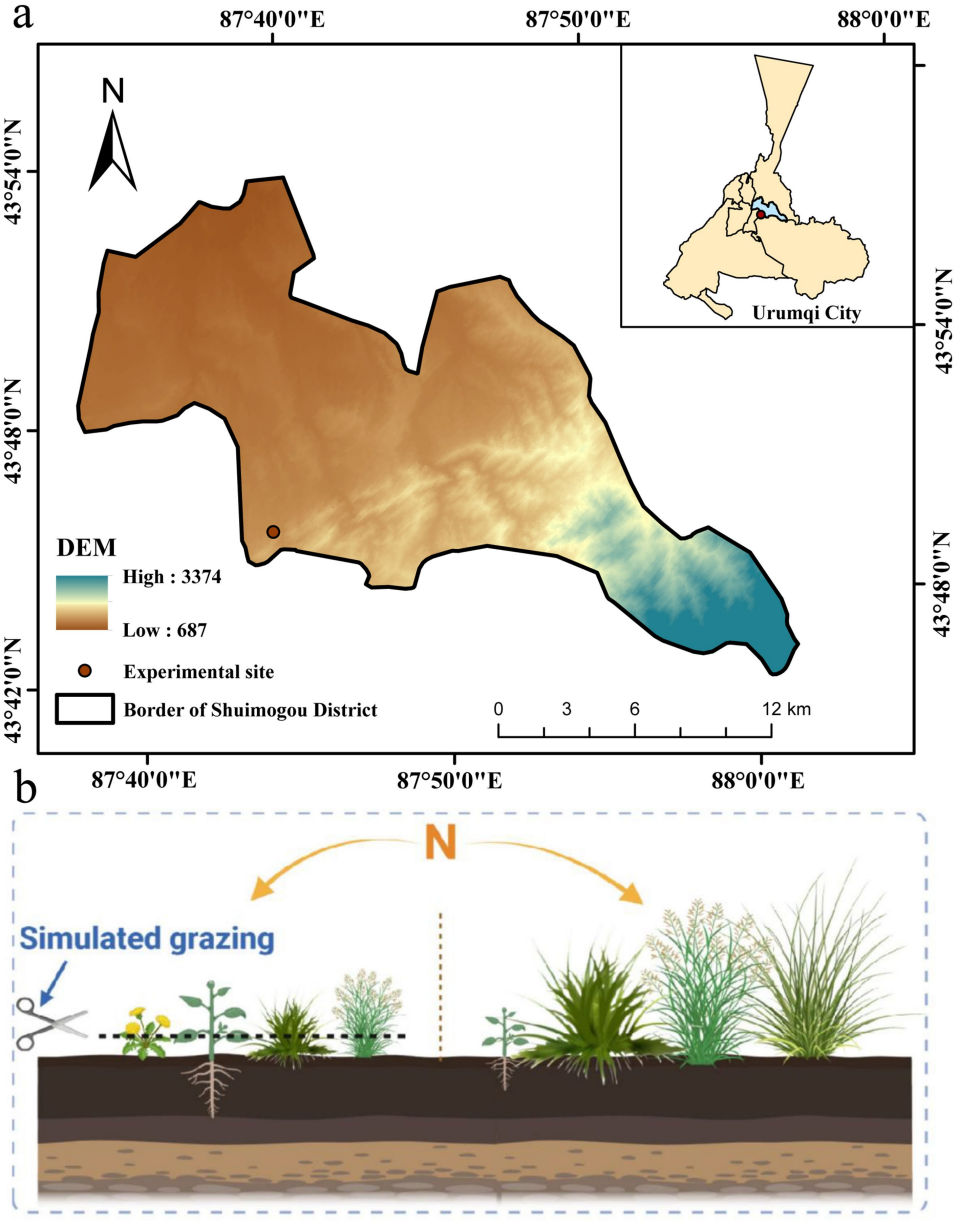
**(a)** Elevation map showing the location of the study area; **(b)** Experimental layout within the plot.

The study area is located at the Yushugou Grassland Monitoring Station in the Shuimogou District of Urumqi (E: 87° 42′, N: 43° 46′), with an elevation of 1,010 m (Figure 1a). The area lies deep in the continental interior, belonging to the temperate continental arid climate zone. The spring and autumn seasons are short, while the winter and summer seasons are long. The region experiences large diurnal temperature variation and extreme seasonal temperature fluctuations, with an average annual precipitation of 236 mm, an average annual temperature of 7.3 °C, and an annual evaporation rate of 2,300 mm. The grassland type is classified as temperate desert steppe, with dominant species including Kentucky bluegrass (*Poa pratensis*), Sarpan needlegrass (*Stipa sareptana*), and Ili sagebrush (*Seriphidium transiliense*). Associated species include prostrate kochia (*Kochia prostrata*), woody milkvetch (Astragalus arbuscula), sheep fescue (*Festuca ovina*), prairie sedge (*Carex liparocarpos*), and aromatic ziziphora (*Ziziphora clinopodioides*).

### 2.2 Experimental design

The experiment was designed as a split-zone experiment, with sample area set up in 2022, the main zone for nitrogen application (N), and the secondary zone for simulated grazing (SG) (Figure 1b), and the nitrogen additions were referenced to the historical nitrogen deposition in Urumqi, which was 28.7 kg/(hm^2^-a) in the urban area and the outskirts of the city (Liang et al., 2018). Nitrogen application treatments were carried out for 2 consecutive years, with nitrogen applied twice a year, half of which was in late April and half in late May, and six different rates of nitrogen application were set: N0: 0 g/m^2^; N1: 2.5 g/m^2^; N2: 5.0 g/m^2^; N3: 10.0 g/m^2^; N4: 15.0 g/m^2^; and N5: 20.0 g/m^2^, with nitrogen fertilizer as urea (46% N). It was spread twice a year. Five replicates were set up for each treatment, with a total of 30 plots, each measuring 4 m × 5 m. The plots were spaced at least 0.5 m apart. For each N application, a weighed amount of urea was dissolved in 5 L of water and applied to the sample plots using a portable spray can. 5 L of water without urea was also applied to the N0 sample plots. Each plot was divided into two sub-plots, simulated grazing and control, and mowed to simulate grazing at the end of August each year when aboveground vegetation reached its peak, leaving stubble at a height of 5 cm after mowing (Jiang et al., 2020).

### 2.3 Soil sampling and measurement methods

Soil sampling was conducted within the plots in August 2023. Soil cores were extracted at a depth of 0–10 cm using a soil auger. Three sampling points were selected diagonally opposite each other within each plot. Soil samples from these three points were thoroughly mixed to form a single composite sample, which was placed in a labeled sealed bag and transported back to the laboratory. A portion of the samples was refrigerated at 4 °C for determining ammonium nitrogen, nitrate nitrogen, and soil enzyme activities (β-1,4-glucosidase, N-acetyl-β-D-glucosidase, Leucine aminopeptidase, Alkaline phosphatase). The remaining portion is naturally air-dried indoors after removing plant roots, stones, and other debris. The soil sample is then ground, mixed thoroughly, and sieved through 1 mm and 0.25 mm screens for storage. This sieved material is used for determining soil pH, electrical conductivity, soil bulk density, soil organic carbon, total nitrogen, total phosphorus, and available phosphorus.

Soil pH was measured using a pH meter with a water-to-soil ratio of 5:1. Soil electrical conductivity (EC) was determined with a conductivity meter at the same water-to-soil ratio (5:1). Soil moisture content was determined by oven-drying at 105 °C for 24 h, and soil bulk density was measured using the core method. Soil organic carbon, total nitrogen, total phosphorus, ammonium nitrogen, nitrate nitrogen, and available phosphorus were determined using the external heating potassium dichromate oxidation method, the Kjeldahl method, the HClO_4_–H_2_SO_4_ digestion followed by the molybdenum–antimony colorimetric method, the indophenol blue spectrophotometric method, the spectrophotometric method, and the 0.5 mol L^−1^ NaHCO_3_ extraction followed by the molybdenum–antimony colorimetric method, respectively. Enzyme activity assays were conducted using kit methods: Soil carbon acquisition enzyme: β-1,4-glucosidase (βG) kit (JC0904-M); Soil nitrogen acquisition enzymes: N-acetyl-β-D-glucosidase (NAG) kit (JC0933-M), Leucine aminopeptidase (LAP) kit (JC0928-M); Soil phosphorus acquisition enzymes: Alkaline phosphatase (ALP) kit (JC0917-M).

### 2.4 Soil microbial sample collection and analysis methods

Soil microbial samples were collected from the 0–10 cm soil layer within each quadrat. The samples from each quadrat were thoroughly homogenized, sealed in sterile bags, and transported to the laboratory in a vehicle-mounted freezer at −20 °C. The procedures for analyzing soil microbial diversity were as follows: (1) Total genomic DNA was extracted from soil samples using a metagenomic DNA extraction kit. (2) The V3–V4 hypervariable region of the bacterial 16S rRNA gene (and the ITS1-1F region of the fungal ITS rRNA gene) was amplified using barcoded primers 338F–806R for bacteria and ITS1-1F-F/ITS1-1F-R for fungi. PCR amplification was performed to obtain the target amplicons. (3) The PCR products were quantified and used for library construction, followed by high-throughput sequencing on the Illumina MiSeq PE300 platform (Illumina, USA) to obtain the nucleotide sequence data of bacterial and fungal variable regions. (4) Sequencing reads were processed and clustered into operational taxonomic units (OTUs) using the QIIME 2.0 software package (QIIME 2 Consortium, USA). Representative OTU sequences were aligned against the SILVA database for bacteria and the UNITE database for fungi to obtain taxonomic assignments (from phylum to species levels) and corresponding abundance information. (5) Diversity indices, including Chao1, Shannon, and Simpson, were calculated to evaluate the richness and evenness of bacterial and fungal communities in the samples.

### 2.5 Statistical analyses

Statistical analysis was performed using SPSS 26.0 (IBM Corporation, USA) to conduct one-way analysis of variance (ANOVA) and independent sample *t*-tests on the soil physicochemical properties and microbial α-diversity between the control and simulated grazing area. The β-diversity of microbial communities was estimated using the Bray-Curtis distance in the “vegan” package and visualized with the “ggplot2” package in R version 4.4.2. Based on Spearman correlation analysis, we employed the “corr.test” function from the “psych” package in R to construct microbial co-occurrence networks for each treatment. To mitigate the influence of rare taxa, only operational taxonomic units (OTUs) present in the samples and meeting the minimum abundance threshold were retained for network construction. Statistical robustness of correlations was determined when the absolute Spearman correlation coefficient exceeded 0.8 and the Benjamini-Hochberg-corrected *p*-value was less than 0.01 (Benjamini and Hochberg, 1995). In the co-occurrence networks constructed from the resulting adjacency matrices, nodes represented microbial taxa, and edges indicated significant correlations between taxa. Gephi (Gephi Alliance, France) was used for visualization and to calculate network topological properties (average degree, modularity index, average clustering coefficient, and average network distance) to assess network complexity and stability (Yang et al., 2024). The random forest model was applied using the “randomForest” package. The Mantel test was conducted using the “linkET” package in R version 4.4.2, and a structural equation model (SEM) to explore the relationship between soil properties and microbial communities was constructed using the “lavaan” package in R version 4.4.2. All graphs were generated using Origin 2021 (OriginLab Corporation, USA), and data are presented as mean ± standard error (SE).

## 3 Results

### 3.1 Effects of nitrogen addition and simulated grazing on microbial diversity

**Figure 2:**
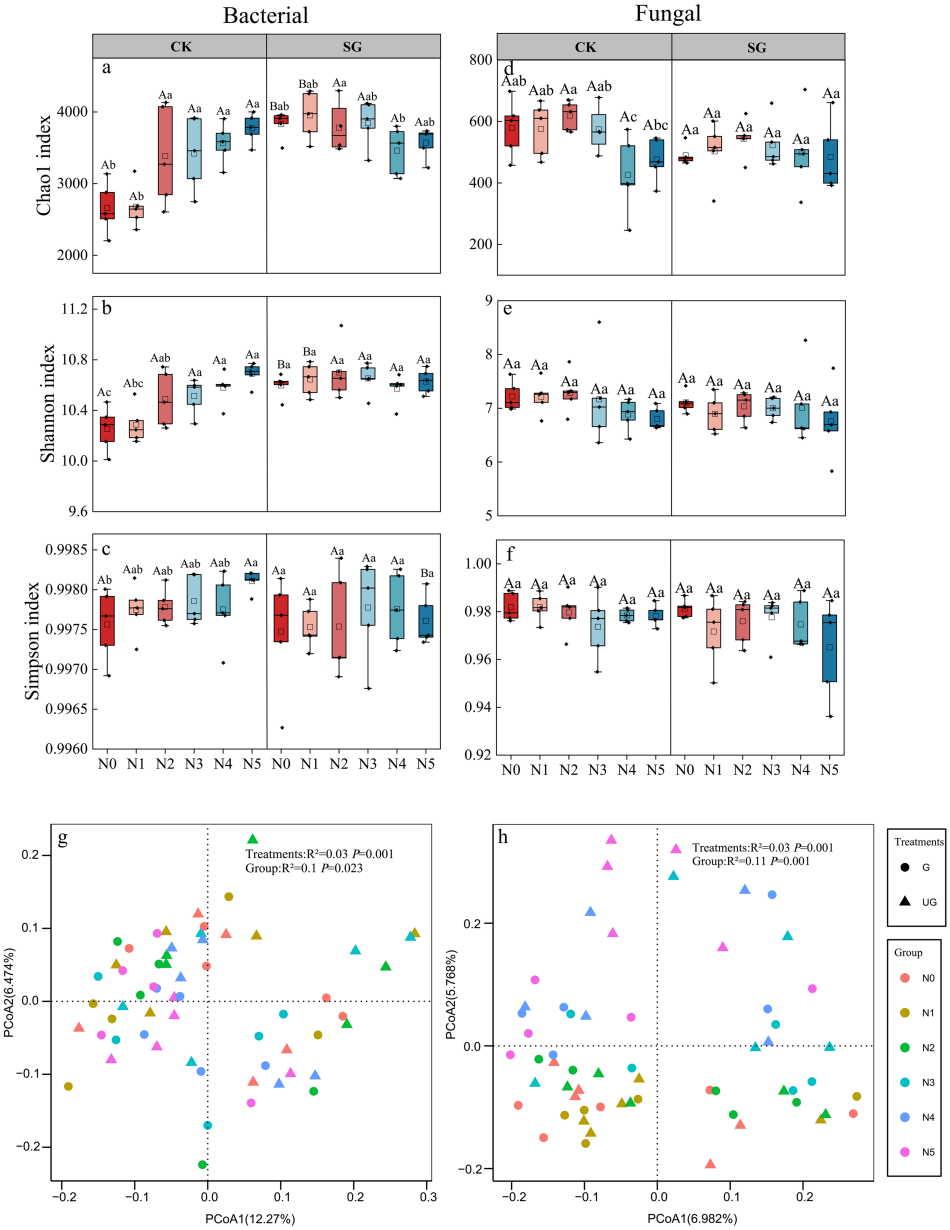
Effects of nitrogen addition and simulated grazing on microbial diversity. CK: Control area; SG: Simulated grazing area; Different capital letters indicate significant differences between simulated grazing and control treatments under the same nitrogen application treatment (*p* < 0.05); Different lowercase letters indicate significant differences in nitrogen addition levels among the same treatments (*p* < 0.05). **(a–c)** Effect of nitrogen addition and simulated grazing on bacterial Chao1 index, Shannon index, and Simpson index; (d–f) Effect of nitrogen addition and simulated grazing on fungal Chao1 index, Shannon index, and Simpson index; (g) Effects of nitrogen addition and simulated grazing on bacterial beta diversity; (h) Effects of nitrogen addition and simulated grazing on fungal beta diversity.

The effects of nitrogen addition and simulated grazing on microbial α-diversity are shown in Figure 2. For bacteria, Chao1 index significantly increased by 27.20% under N2 treatment compared to N0 in the control area, whereas excessively high levels of applied nitrogen (N3–N5) showed no significant change compared to N2 instead, suggesting that moderate nitrogen inputs may increase species richness, but high nitrogen triggers a threshold effect. In the simulated grazing area, the Chao1 index showed a significant increase followed by a decline, and the values under N0 and N1 treatments were significantly higher than those in the control area by 43.92 and 47.57%, respectively (*p* < 0.05; Figure 2a).

### 3.2 Effects of nitrogen addition and simulated grazing on microbial co-occurrence networks

**Figure 3:**
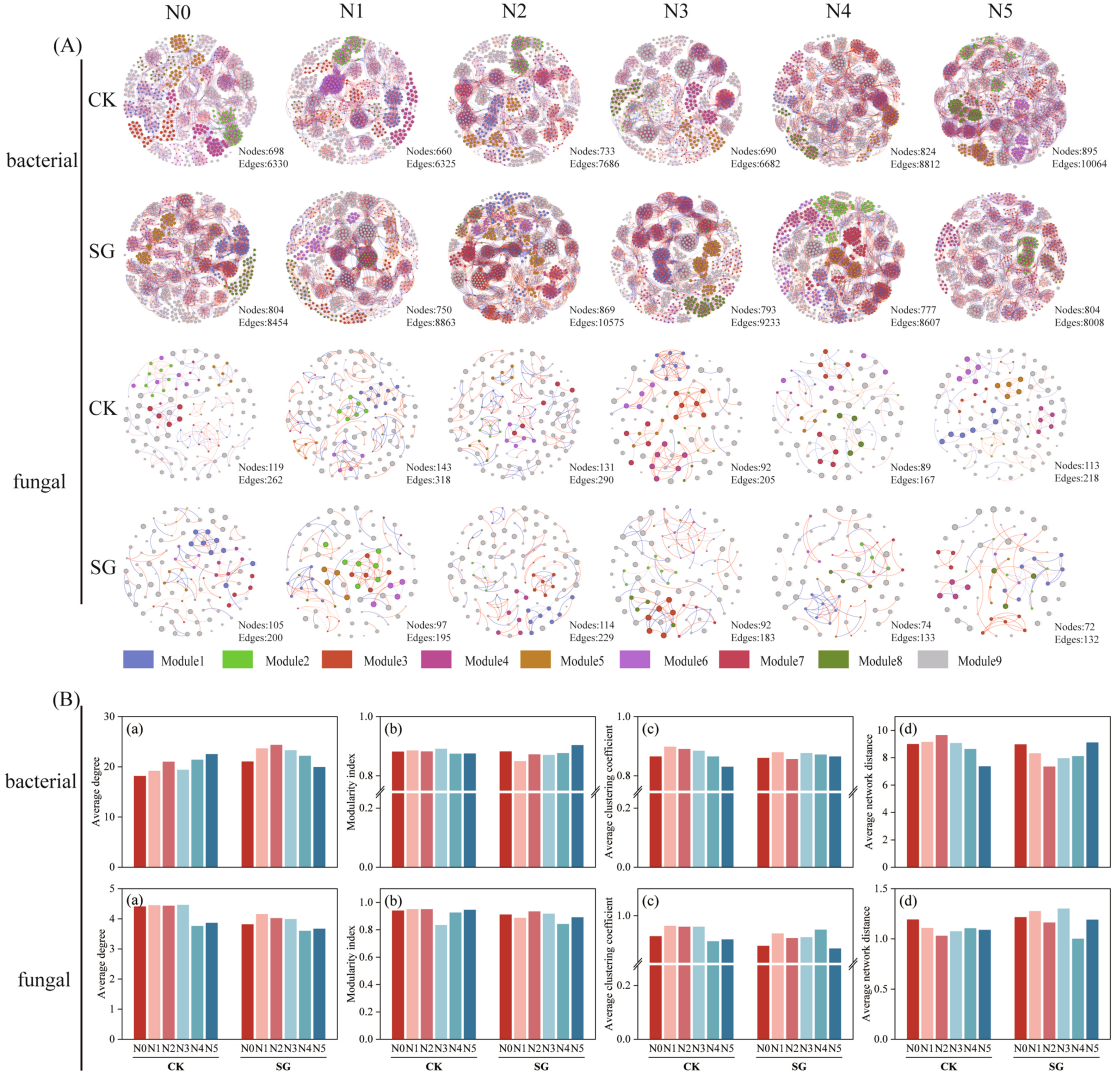
Effects of nitrogen addition and simulated grazing on microbial co-occurrence networks and network topology coefficients. **(A)** Microbial co-occurrence networks; **(B)** network topology coefficients; **(a)** Average degree of bacteria and fungi; **(b)** modularity index of bacteria and fungi; **(c)** average clustering coefficient of bacteria and fungi; **(d)** average network distance of bacteria and fungi. CK: Control area; SG: simulated grazing area.

Although the Shannon and Simpson indices for bacteria in the control area reached high values under high nitrogen (N5), this does not imply greater system stability. *α*-diversity is a “compositional attribute” whereas network complexity and topological properties more directly determine system stability and vulnerability. By analyzing the response of soil microbial co-occurrence networks to nitrogen addition and simulated grazing (Figure 3), the results show that, both in the control and simulated grazing area, the network parameters of the soil bacterial communities exhibited higher complexity, specifically manifested as more nodes and edges. In contrast, soil fungal communities in the simulated grazing area exhibited fewer nodes and edges than those in the control area (Figure 3A). In bacterial communities, the modularity index, average clustering coefficient, and average network distance in the control area all exhibited a trend of initially increasing followed by a decrease. However, in simulated grazing areas, the topological coefficients of bacterial networks exhibited an opposite trend, showing a certain degree of enhancement. For fungal communities, the network complexity in the simulated grazing area (such as average degree, modularity index, and average clustering coefficient) was generally lower than in the control area, while the average network distance was generally higher than in the control area (Figure 3B).

### 3.3 Main factors influencing soil microbial diversity

**Figure 4:**
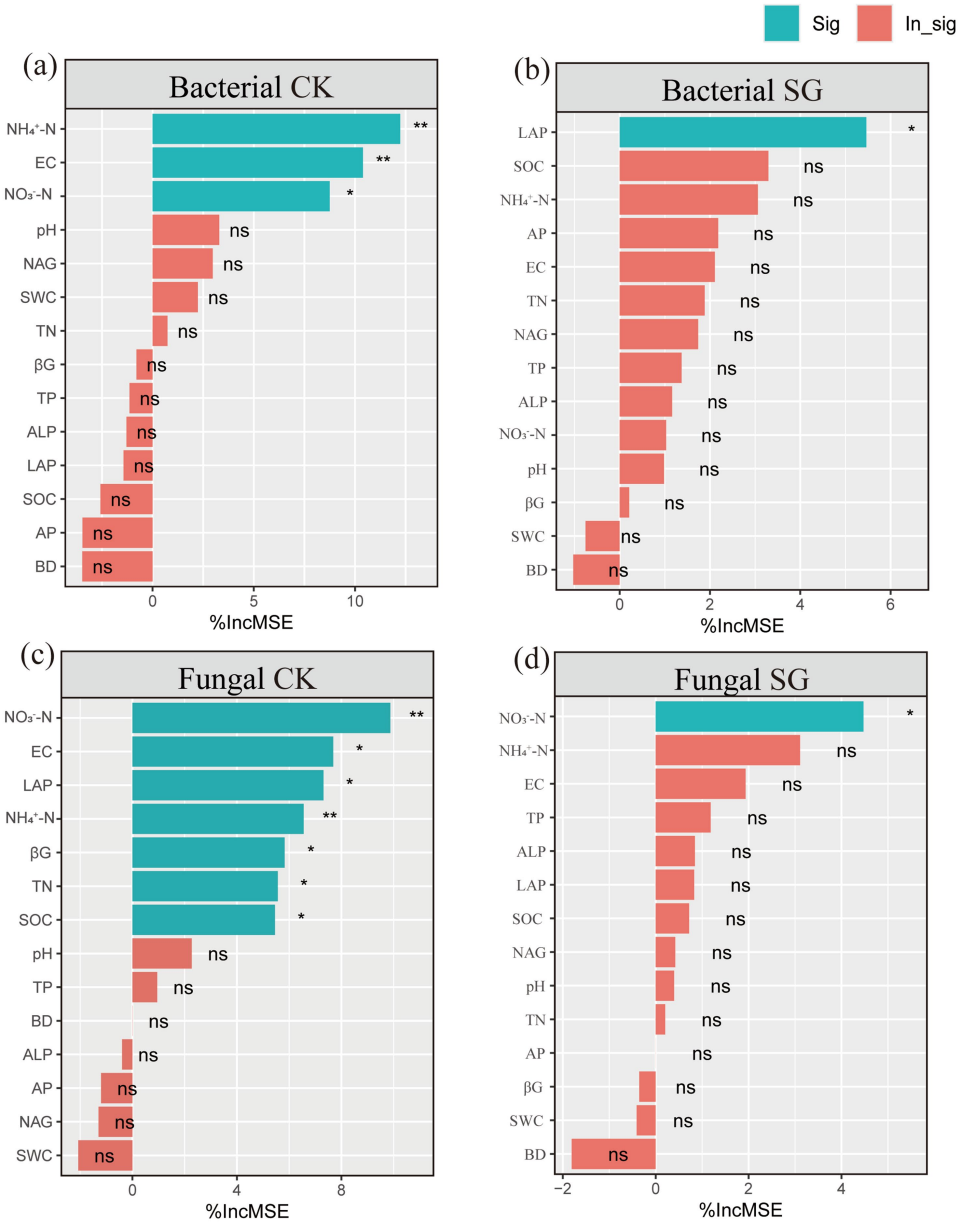
Ranking of main influencing factors on bacterial and fungal diversity. **(a)** Ranking of environmental factors affecting bacterial diversity in the control area; **(b)** Ranking of environmental factors affecting bacterial diversity in the simulated grazing area; **(c)** Ranking of environmental factors affecting fungal diversity in the control area; **(d)** Ranking of environmental factors affecting fungal diversity in the simulated grazing area. “*,” “**,” and “***” indicate significant differences (*p* < 0.05), highly significant differences (*p* < 0.01) and (*p* < 0.001) in environmental factors, while “ns” indicates no significant difference (*p* > 0.05); CK: Control area; SG: Simulated grazing area; SWC: Soil moisture content; BD: Bulk density; pH: Soil acidity and alkalinity; EC: Soil electrical conductivity; SOC: Soil organic carbon; TN: Total nitrogen; TP: Total phosphorus; NO_3_^−^-N: Nitrate nitrogen; NO_4_^+^-N: Ammonium nitrogen; AP: vailable phosphorus; βG: β-1,4-lucosidase; ALP: lkaline phosphatase; NAG: N-acetyl-β-D-glucosidase; LAP: Leucine aminopeptidase.

The results of the random forest importance ranking (Figure 4) show that the main factors influencing microbial diversity in the control area are inorganic nitrogen (nitrate nitrogen and ammonium nitrogen). In the simulated grazing area, the main factor influencing bacterial diversity is LAP activity; The main factor influencing fungal diversity is nitrate nitrogen.

### 3.4 Response pathways of microbial diversity to nitrogen addition and simulated grazing

**Figure 5:**
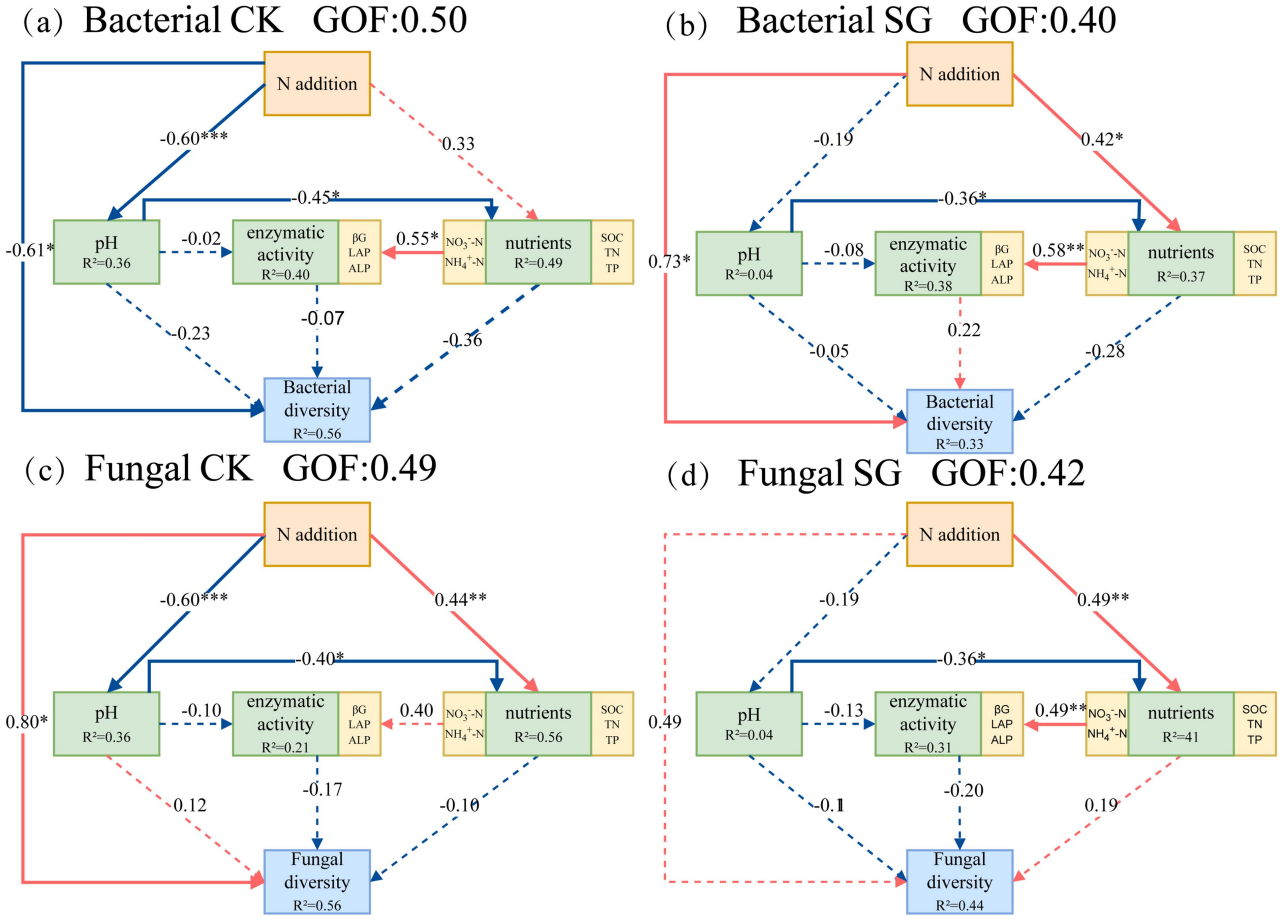
The impact pathways of nitrogen addition on bacterial and fungal diversity in control and simulated grazing areas. CK: Control area; SG: Simulated grazing area; “*,” “**,” and “***” respectively indicated significant (*p* < 0.05), extremely significant (*p* < 0.01), and (*p* < 0.001); **(a)** SEM of bacteria in the control area; **(b)** SEM of bacteria in the simulated grazing area; **(c)** SEM of fungi in the control area; **(d)** SEM of fungi in the simulated grazing area.

Partial Least Squares Path Modeling (PLS-PM) was used to identify the complex multivariable relationships between soil and climate parameters, crop rotation, agricultural intensity, and soil microbial communities. Based on the interpretation of the coefficient of determination (*R*^2^) and goodness of fit (GOF), generally, when the GOF is greater than 0.4, the model is considered acceptable, and when the GOF exceeds 0.7, the model is considered excellent (Dunn et al., 2021). Path coefficients represent the direction and strength of the direct effects between two variables (Li et al., 2019). The PLS-PM model was used to analyze the direct and indirect effects of nitrogen addition, pH, soil nutrients, enzyme activity, and bacterial and fungal diversity in the control and simulated grazing area (Figure 5). In the bacterial control area, the GOF of the PLS-PM model was 0.50, and nitrogen addition had a significant negative direct effect on bacterial diversity (*p* < 0.05), with a path coefficient of −0.61. In the bacterial simulated grazing area, the GOF of the PLS-PM model was 0.40, and nitrogen addition had a significant positive direct effect on bacterial diversity (*p* < 0.05), with a path coefficient of 0.73. This suggests that simulated grazing alleviated the negative impact of nitrogen addition on bacterial diversity. In the fungal control area, the GOF of the PLS-PM model was 0.49, and nitrogen addition had a significant positive direct effect on fungal diversity (*p* < 0.05), with a path coefficient of 0.80. In the fungal simulated grazing area, the GOF of the PLS-PM model was 0.42, and nitrogen addition had a positive direct effect on fungal diversity, although it was not significant (*p* > 0.05), with a path coefficient of 0.49. It can be seen that the path coefficient for the effect of nitrogen addition on fungal diversity was lower in the simulated grazing area compared to the control area, indicating that simulated grazing partially suppressed the impact of nitrogen addition on fungal diversity.

## 4 Discussion

### 4.1 Responses of microbial co-occurrence networks to nitrogen addition and simulated grazing

The abundance and diversity of microbial communities reflect the composition of soil microbial communities, but are not sufficient to reveal the functional characteristics and mechanisms of microbial action (Qiu and Cardinale, 2020). Previous studies have indicated that microbial communities constitute complex systems composed of highly interacting groups, whose functional outputs are often driven by dynamic associative networks formed among these groups (Wagg et al., 2019). Thus, relying solely on microbial species and diversity fails to adequately explain the significance of microbial interaction mechanisms (Xu et al., 2023). In this context, microbial co-occurrence network analysis has become a key tool for analyzing the regulatory mechanisms of community functions by revealing potential synergistic or competitive relationships among microbial species (Zhang et al., 2024b; Ye et al., 2021). Studies have shown that soil physicochemical properties altered by nitrogen addition and grazing disturbance play a key role in shaping microbial interaction networks (Li and Li, 2025; Ding et al., 2023). Changes in pH and inorganic nitrogen availability driven by external factors alter microbial competitive relationships, thereby influencing the complexity and stability of microbial networks (Coyte et al., 2015). Studies have shown that the more connections (nodes and edges) there are in a soil microbial community network, the more stable it is and the better it is at inhibiting pathogen invasion (Wei et al., 2018). This study found that soil bacterial communities in both the control area and the simulated grazing area exhibited higher network complexity (with more nodes and edges), indicating that bacteria possess stronger resilience to external disturbances and ecological dominance within the temperate desert steppe ecosystem. This result is consistent with the findings of Liu et al. (2024), further indicating that bacterial communities play a central role in nutrient cycling and ecological network stability. Network topology coefficients reflect environmental fluctuations and community stability in ecosystems and are indicators of ecosystem adaptation to different environmental conditions (Lupatini et al., 2014; Jia et al., 2021). Consistent with the first hypothesis, the nonlinear effect of nitrogen addition on soil bacterial community diversity. In this study, the trend of increased bacterial diversity in the control area under high nitrogen treatment became less pronounced. Furthermore, the modularity index, average clustering coefficient, and average network distance of bacterial networks in the control area exhibited a trend of initially increasing and then decreasing with nitrogen addition. This suggests that moderate nitrogen addition may promote functional differentiation and stability within bacterial networks, but excessive nitrogen input disrupts the equilibrium of the original network structure. This is consistent with the findings of Wang et al. (2024a), indicating that nitrogen addition levels are a key factor influencing ecological network relationships. In contrast, the bacterial network in the simulated grazed area showed the opposite response pattern, possibly because grazing reduces aboveground biomass and enhances plant demand for available nitrogen, thereby increasing plant dependence on microbial-mediated nutrient acquisition processes (Singh et al., 2022; Ma et al., 2024). This suggests that enzyme-mediated nitrogen transformations may promote more efficient resource utilization by bacterial communities, thereby increasing network complexity and stability under high nitrogen addition conditions. Unlike bacteria, fungal communities showed a trend of reduced network complexity (fewer nodes and edges) in the simulated grazing area and generally lower mean degree, modularity index and mean clustering coefficient than in the control area, whereas the mean path lengths increased, suggesting that the fungal community network was looser and less stable. It is possible that grazing induced changes in soil physicochemical properties such as reduced soil pH and increased nitrate nitrogen may have weakened the ecological stability and interactions of the fungal community (Ding et al., 2023).

### 4.2 Responses of microbial diversity to nitrogen addition and simulated grazing

**Figure 6:**
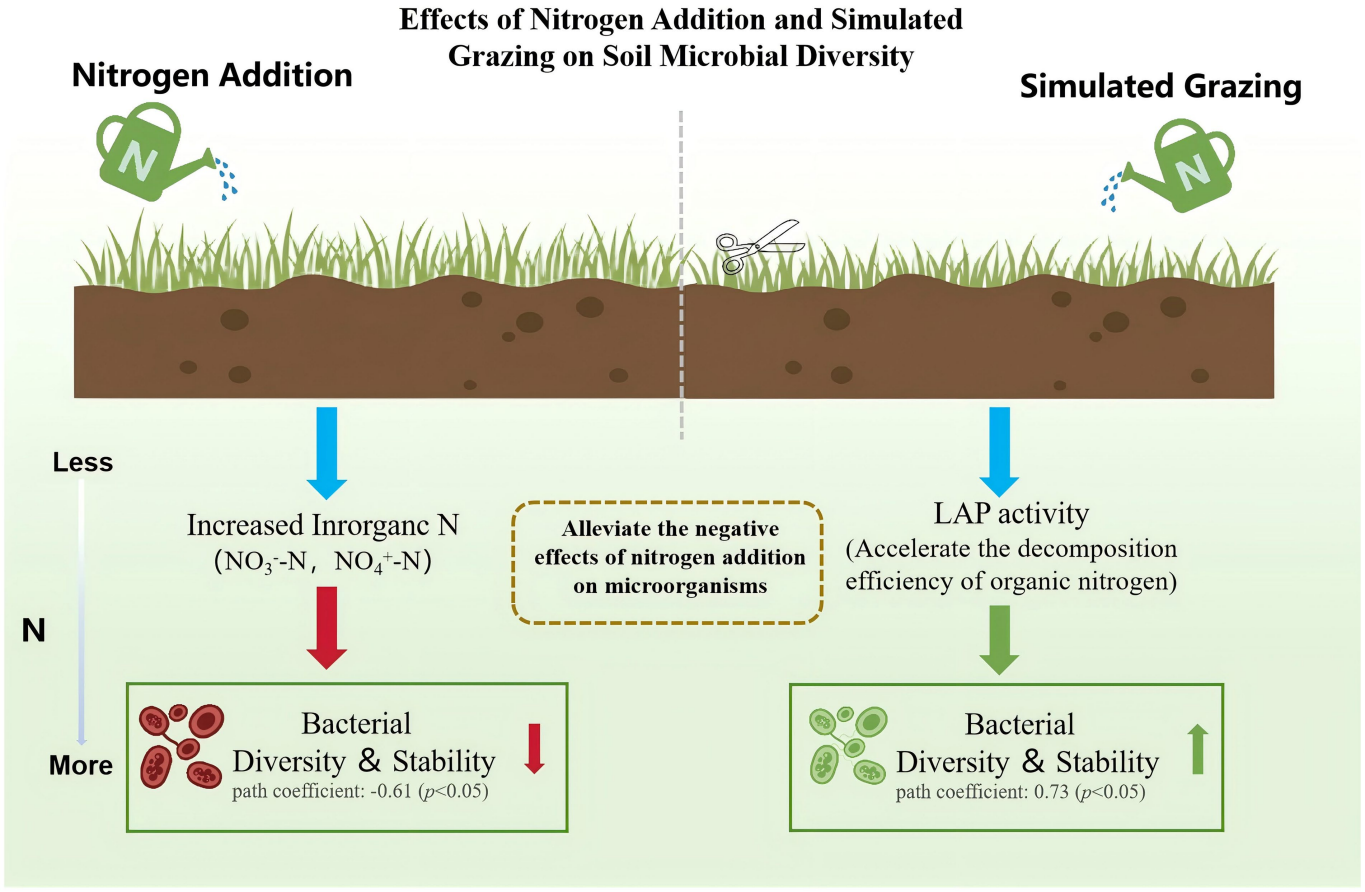
Grazing can mitigate the adverse effects of nitrogen addition on soil microorganisms.

Soil inorganic nitrogen and enzyme activities acted as key mediators linking nitrogen addition and grazing to microbial diversity (Figure 4). To further elucidate the mechanisms underlying microbial community responses, random forest importance ranking and partial least squares path modeling (PLS-PM) were used to analyze the effects of soil environmental factors on bacterial and fungal diversity in the control and simulated grazing areas. The results showed that in the control area, nitrate nitrogen and ammonium nitrogen jointly determined bacterial and fungal diversity (Figures 4a,c), indicating that nitrogen addition directly or indirectly altered microbial community structure by changing soil nutrient availability (e.g., increasing nitrate and ammonium contents) (Zhang et al., 2024a; Song et al., 2025). This finding supports the second hypothesis that available inorganic nitrogen is a key factor regulating microbial community structure. The possible explanation is that nitrogen addition provides sufficient nitrogen substrates for nitrifying and denitrifying bacteria, thereby stimulating the activity of microorganisms involved in the nitrogen cycle (Liang and Robertson, 2021). Compared with the control area, bacterial diversity in the simulated grazed area was mainly influenced by LAP activity, which may be closely related to the intensified N competition between plants and microorganisms under grazing disturbance. Grazing limited direct microbial access to NO_4_^+^-N and NO_3_^−^-N by reducing the supply of inorganic nitrogen directly available in the soil through lowering aboveground biomass, promoting rapid plant regeneration, and enhancing competition for inter-root resources. In this inorganic nitrogen-limited environment, bacteria prefer to accelerate the process of organic nitrogen catabolism by enhancing extracellular enzyme activity in order to obtain inorganic nitrogen needed to maintain community function and growth (Zhu et al., 2025). By contrast, the drivers of fungal diversity under grazing conditions were more singular, being significantly correlated only with nitrate nitrogen. This suggests that grazing may alter soil structure and nutrient conditions, thereby weakening the fungal community’s responsiveness to multiple environmental factors and enhancing its dependence on nitrate nitrogen (Zhang et al., 2024c). PLS-PM analysis further revealed that nitrogen addition had a significant negative effect on bacterial diversity in the control area (path coefficient = −0.67), whereas it exerted a significant positive effect in the simulated grazing area (path coefficient = 0.76). This is consistent with the third hypothesis that grazing may mitigate to some extent the negative effects of nitrogen addition on microbial communities. This may be due to increased competition for dominant microbial taxa as a result of nitrogen fertilizer enrichment, but moderate grazing mitigated the negative effects of nitrogen fertilizer additions on the stability of the bacterial community by regulating the competitive relationships between dominant bacterial taxa.(Li et al., 2024). Regarding fungal diversity, nitrogen addition in the control area produced a significant positive effect (path coefficient = 0.94), whereas in the simulated grazing area, it weakened the positive effect it generated (path coefficient = 0.46). This may be attributed to grazing enhancing plant uptake of available phosphorus, thereby reducing microbial access to phosphorus and limiting microbial biomass production, which indirectly suppressed (Bai et al., 2021). Overall, the results indicate that grazing mitigates the adverse effects of nitrogen addition on microbial communities to some extent by influencing nitrogen availability, enzyme-mediated nutrient acquisition processes, and microbial interaction structures (Figure 6). This study deepens our understanding of the response mechanisms of soil microorganisms in temperate desert grasslands under multiple external stressors. It demonstrates that under the context of continuously increasing nitrogen deposition, rational grazing management plays a crucial role in maintaining the stability of microbial functions.

## 5 Conclusion

This study systematically revealed the combined effects of nitrogen fertilization at different concentrations and simulated grazing on microbial community diversity and stability through experiments conducted in warm-temperate desert grasslands. The results indicate that inorganic nitrogen is the key factor driving nitrogen-induced changes in microbial diversity. Adequate nitrogen addition increased bacterial community diversity, but excessive nitrogen addition weakened this positive effect and reduced bacterial community stability, while moderate grazing mitigated these negative effects by enhancing LAP activity to promote organic nitrogen decomposition and regulating resource competition. This study reveals the interaction mechanism between nitrogen addition and grazing, emphasizes the important role of grazing in maintaining microbial diversity and functional stability of temperate desert grassland ecosystems, and provides a scientific basis for the sustainable management of grassland ecosystems in the context of global change.

## Data Availability

The raw data supporting the conclusions of this article will be made available by the authors, without undue reservation.
