# Supplementary material for: Nitrogen addition has negative effects on bacterial diversity and stability, but simulated grazing mitigates these effects
Source: Front Microbiol. 2026 Feb 17;17:1747173. doi: 10.3389/fmicb.2026.1747173 (PMC12953565; doi:10.3389/fmicb.2026.1747173)
Supplement: Supplementary file 1 [file Table_1.docx]

**Table S1** Effects of nitrogen addition and simulated grazing on soil physicochemical properties

| Treatment | | pH | conductivity  (ms·cm^-1^) | capacity  (g·cm^-3^) | moisture content  (%) | Soil organic carbon  (g·kg^-1^) | Total nitrogen  (g·kg^-1^) | Total phosphorus  (g·kg^-1^) | Nitrate  (mg·kg^-1^) | Ammonium  (mg·kg^-1^) | Available phosphorus  (g·kg^-1^) |
| --- | --- | --- | --- | --- | --- | --- | --- | --- | --- | --- | --- |
|  |  |  |  |  |  |  |  |  |  |  |  |
| CK | N0 | 7.68±0.10Aa | 121.06±7.18Ad | 1.14±0.05Aa | 6.81±0.35Aa | 22.75±1.52Aa | 2.27±0.13Aab | 0.85±0.02Aa | 6.90±0.68Ad | 4.80±0.84Abc | 6.26±0.32Aa |
|  | N1 | 7.56±0.09Aab | 140.72±11.24Acd | 1.12±0.04Aa | 7.19±0.28Aa | 22.83±1.58Aa | 2.15±0.12Ab | 0.88±0.02Aa | 13.77±2.81Acd | 4.09±0.20Ac | 8.12±0.87Aa |
|  | N2 | 7.49±0.09Aabc | 156.78±14.13Abc | 1.11±0.05Aa | 7.07±0.33Aa | 27.47±2.35Aa | 2.66±0.21Aa | 0.89±0.01Aa | 21.42±4.52Ac | 5.07±0.26Abc | 6.14±0.78Aa |
|  | N3 | 7.50±0.05Aabc | 187.98±12.21Ab | 1.15±0.02Aa | 7.33±0.17Aa | 25.18±2.27Aa | 2.50±0.19Aab | 0.87±0.01Aa | 51.86±6.96Ab | 10.05±1.33Aab | 6.04±0.33Aa |
|  | N4 | 7.43±0.03Abc | 184.70±8.77Ab | 1.14±0.04Aa | 6.74±0.24Aa | 23.47±1.36Aa | 2.43±0.13Aab | 0.90±0.02Aa | 54.87±4.23Ab | 11.45±3.24Aa | 6.54±0.74Aa |
|  | N5 | 7.26±0.09Ac | 226.20±7.00Aa | 1.22±0.02Aa | 6.93±0.71Aa | 21.69±1.4Aa | 2.25±0.14Aab | 0.87±0.02Aa | 80.81±3.81Aa | 13.62±2.19Aa | 7.46±0.80Aa |
| SG | N0 | 7.49±0.11Aa | 129.98±5.35Ad | 1.15±0.03Aa | 6.67±0.19Aab | 24.02±2.02Aa | 2.36±0.16Aa | 0.91±0.02Aa | 9.23±0.81Ac | 4.00±0.38Ab | 7.16±0.46Aab |
|  | N1 | 7.60±0.05Aa | 130.12±5.59Ad | 1.16±0.06Aa | 7.33±0.37Aa | 22.22±2.35Aa | 2.18±0.22Aa | 0.90±0.02Aa | 15.03±1.76Ac | 3.97±0.21Ab | 6.32±0.21Aab |
|  | N2 | 7.58±0.04Aa | 155.22±6.51Acd | 1.21±0.04Aa | 6.78±0.30Aab | 23.36±1.61Aa | 2.35±0.16Aa | 0.89±0.01Aa | 24.68±3.71Ac | 6.05±0.96Ab | 5.36±0.55Ab |
|  | N3 | 7.54±0.07Aa | 171.88±8.74Abc | 1.15±0.04Aa | 6.03±0.53Ab | 25.59±2.71Aa | 2.44±0.18Aa | 0.90±0.03Aa | 42.19±5.46Ab | 7.85±1.19Ab | 5.36±0.5Ab |
|  | N4 | 7.51±0.05Aa | 184.32±8.22Ab | 1.20±0.03Aa | 6.84±0.21Aab | 23.86±1.41Aa | 2.46±0.13Aa | 0.91±0.02Aa | 56.70±5.79Ab | 25.88±5.65Aa | 7.24±0.59Aa |
|  | N5 | 7.46±0.06Aa | 230.68±16.84Aa | 1.17±0.03Aa | 7.15±0.40Aab | 21.55±1.06Aa | 2.31±0.11Aa | 0.92±0.01Aa | 82.86±9.18Aa | 32.68±10.93Aa | 7.56±0.90Aa |

Note: CK: Control area；SG: Simulated grazing area; Different capital letters indicate significant differences between simulated grazing and control treatments under the same nitrogen application treatment (*p*<0 05); Different lowercase letters indicate significant differences between the same treatments (*p*<0 05).

**Figure S1** Effects of nitrogen addition and simulated grazing on soil enzyme activity**.** CK: Control area；SG: Simulated grazing area; Different capital letters indicate significant differences between simulated grazing and control treatments under the same nitrogen application treatment (*p* < 0 05); Different lowercase letters indicate significant differences between the same treatments (*p* < 0 05).
